# The SMILE study: a study of medical information and lifestyles in Eindhoven, the rationale and contents of a large prospective dynamic cohort study

**DOI:** 10.1186/1471-2458-8-19

**Published:** 2008-01-21

**Authors:** Marjan van den Akker, Mark G Spigt, Lore De Raeve, Ben van Steenkiste, Job FM Metsemakers, Ernst J van Voorst, Hein de Vries

**Affiliations:** 1 Department of General Practice, School for Public Health and Primary Care (CAPHRI), Maastricht University, Maastricht, The Netherlands; 2 Department of Epidemiology, School for Public Health and Primary Care (CAPHRI), Maastricht University, Maastricht, The Netherlands; 3 The Eindhoven Corporation of Primary Health Care Centers, Eindhoven, The Netherlands; 4 Department of Health Education and Promotion, School for Public Health and Primary Care (CAPHRI), Maastricht University, Maastricht, The Netherlands

## Abstract

**Background:**

Health problems, health behavior, and the consequences of bad health are often intertwined. There is a growing need among physicians, researchers and policy makers to obtain a comprehensive insight into the mutual influences of different health related, institutional and environmental concepts and their collective developmental processes over time.

**Methods/Design:**

SMILE is a large prospective cohort study, focusing on a broad range of aspects of disease, health and lifestyles of people living in Eindhoven, the Netherlands. This study is unique in its kind, because two data collection strategies are combined: first data on morbidity, mortality, medication prescriptions, and use of care facilities are continuously registered using electronic medical records in nine primary health care centers. Data are extracted regularly on an anonymous basis. Secondly, information about lifestyles and the determinants of (ill) health, sociodemographic, psychological and sociological characteristics and consequences of chronic disease are gathered on a regular basis by means of extensive patient questionnaires. The target population consisted of over 30,000 patients aged 12 years and older enrolled in the participating primary health care centers.

**Discussion:**

Despite our relatively low response rates, we trust that, because of the longitudinal character of the study and the high absolute number of participants, our database contains a valuable set of information.

SMILE is a longitudinal cohort with a long follow-up period (15 years). The long follow-up and the unique combination of the two data collection strategies will enable us to disentangle causal relationships. Furthermore, patient-reported characteristics can be related to self-reported health, as well as to more validated physician registered morbidity. Finally, this population can be used as a sampling frame for intervention studies. Sampling can either be based on the presence of certain diseases, or on specific lifestyles or other patient characteristics.

## Background

Good health is highly valued. For centuries with varying success scientists have tried to find causes of illness and ways to prevent ill health. A lot of progress in improving and maintaining health has been made, especially in the area of infectious diseases. Chronic diseases, however, do not respond to the simple formula "identify the cause and eliminate it" [1], because their development is determined by multiple factors, resulting in limited possibilities for rapid interventions. Even when important risk factors are known, for example smoking in chronic obstructive pulmonary disease, and obesity in diabetes mellitus type II, it appears difficult to intervene in an effective way. Due to the limited success of preventive interventions, changes of the population distribution (a sharp increase of the proportion of elderly people), and societal changes (like changes in social security services, changes in working conditions) the prevalence of chronic diseases is rising, and is expected to rise further in the near future. Given the nature of the current health problems, integral approaches are required, such as advocated by the New Public Health approaches and Ecological Health approaches, where classical medicine collaborates with public health or other disciplines[2,3].

This has resulted in a growing need among physicians, researchers, and policy makers to gain insight into the mutual influences of different health related, institutional and environmental concepts and their collective developmental processes over time. Only substantial insight into these factors and their relations can help to improve public health by effective interventions in the areas of public health, occupational health, disability management, and care in the home. Such insight can be obtained by combining both physician-registered medical data and patient self-reported data. Often, researchers have of either medical data or questionnaire data at their disposal, which both have their limitations.

Health problems and health behavior are often intertwined, and it is therefore relevant to gather both medical data as registered in general practice and health related data as reported by the patients. Although this notion seems trivial, very few studies do so. The collection of this combined data, albeit more complex and expensive, will have several advantages. First a better understanding of health problems can be developed. Secondly, it is possible to evaluate 'natural' occurring phenomena. E.g. the results of a recent study on health problems of victims of a fireworks disaster in the Netherlands could have been more explicit if self-reported data on patients' perception were also available [4].

Moreover, an integral approach collecting medical, health behavioral, psychosocial and care consumption data, has substantial advantages.

The goal of the SMILE study is to develop a hybrid database containing both medical data and questionnaire data, attempting to resolve the problems described above. Although results of outcomes of elements of this study are already described [5-7], this paper describes the methodology and design of this new dynamic cohort in primary care, using both medical and questionnaire data, and aiming at gaining a comprehensive, interdisciplinary and modern insight into the mutual influences of different health related, institutional and environmental factors. The name of this new cohort is The **S**tudy of **M**edical **I**nformation and **L**ifestyles in **E**indhoven, in short SMILE.

### Research model

**Figure 1:**
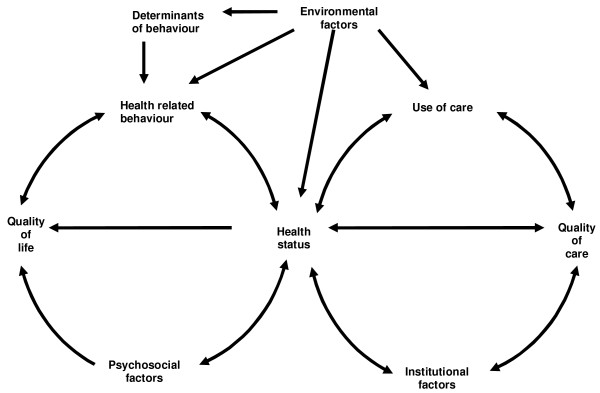
Bicycle model for the SMILE study.

Based on the scientific input of the various disciplines (health education, general practice, medical sociology, health ethics and health philosophy, health economics, epidemiology), cooperating in SMILE, the main points of interest are captured in the research model (see figure 1), called the "bicycle model". Research groups are involved in one or more parts of the model, and, consequently, the different parts of the model are designed and further developed by researchers from one or more disciplines. The SMILE bicycle shows a comprehensive framework with interrelations between different areas of health and health-related research. The two wheels of the bicycle are connected through the "frame" health status. On top of these circles one finds important basic components belonging to the bicycle (saddle and handlebars).

The "front wheel" mainly concerns the patient-related concepts, like health related behavior/lifestyles, quality of life and psychosocial factors. Current lines of research in this wheel focus on the course and consequences of chronic disease, aiming at an increase of insight into the relations between participation, perceived autonomy, health related behavior and the course and consequences of chronic disease.

The "rear wheel" focuses on the institutional aspects of health and health care; use and quality of care, and other institutional factors. In this wheel research regarding continuity and accessibility of care takes place, e.g. aiming at the analysis of the so-called patient careers of chronically ill patients, as well as the influence of culturally determined opinions about health and disease, lifestyles, and values on the use of care and patient careers. Another example of current research taking place in this part of the model concerns the evaluation of a recent major change in health insurance system in the Netherlands and its effects on the quality and accessibility of care as experienced by elderly people.

The "handlebars", containing determinants of behavior, is a main concern from the health educational perspective. Research questions focus on health related behavior, the origin of behavior and results of these behaviors in terms of (changes in) life styles and health. Examples concern the role of parenting styles on the smoking behavior of adolescents [8,7] and factors associated to soft drink consumption and fruit and vegetable intake among adolescents [5,6].

The "saddle" represents the environmental factors. Important research questions from this perspective concern the longitudinal relation between health, health behavior, and their (work-related) determinants among the working and the non-working population, with special attention to psychological complaints and musculoskeletal complaints, in order to be able to analyze the influence of work-related factors on health and health behavior. Another major study from this perspective in combination with the "front wheel" is the influence of socioeconomic status-combined with psychological characteristics on the occurrence of cardiovascular diseases.

A number of research projects have been performed, or are currently being performed using the data available from SMILE. These studies concern: parenting styles and smoking behavior in adolescents; personality and intake of fruit, vegetables and soft drinks in adolescents; inequalities in health perceptions between the employed and the unemployed; clustering of life style behaviors; self-esteem, self-efficacy and depression in adolescent headache sufferers; the influence of life style on the development and prognosis of headache in adolescents; rebelliousness, conformity and socio-economic health differences; material versus psychosocial explanations of socio-economic health differences; change of the health insurance system and quality and accessibility of care; multimorbidity and income in relation to self-reported physical and mental health.

## Methods/Design

SMILE, is a large prospective cohort study, focusing on different aspects of disease, health and lifestyle of people living in Eindhoven, the Netherlands. Eindhoven is a city in the Southern part of the Netherlands of approximately 200,000 inhabitants.

In November 2002, 2,178 adolescents (aged 12–18 years) and 28,879 adults (aged 18 and older) enrolled in one of the participating health care centers were invited to participate in SMILE. SMILE is a dynamic cohort study, meaning that new participants will enter the population sample when they either reach 12 years of age or when they are enrolled as a new patient in one of the participating centers, and they give their informed consent. Respondents may leave the population either through leaving the participating practice or due to death or drop-out. Furthermore, after two succeeding moments of non-respons patients don't receive any more questionnaires. The dynamic character of SMILE is expected to result in a sufficiently large patient population over time and it prevents vanishing of the study population.

The fundamental goal of SMILE is to study health and disease and their determinants and their consequences in a comprehensive approach for an extended period of time (15 years). Apart from serving data for examining and analyzing relationships between several health-related concepts, SMILE can also serve as a sampling frame to select specific patient groups such as elderly with osteoarthritis,, adolescents with low self-esteem, or adults with a sedentary life style.

To realize this goal, the structure and contents of the SMILE study has two unique characteristics:

1. Two data collection strategies are combined: firstly, data on morbidity, mortality, medication, and use of care facilities are continuously registered using electronic medical records (EMRs) in primary health care centers. Secondly, information about lifestyles and the determinants of (ill) health, sociodemographic, psychological and sociological characteristics, and consequences of disease are gathered by means of patient questionnaires.

2. Researchers from various disciplines (health education, general practice, medical sociology, health ethics and health philosophy, health economics, epidemiology), are actively involved in the study. They all contribute to the theoretical framework and the contents of the questionnaire and the analysis of data. As a result different perspectives are explicitly incorporated (see figure 1), resulting in a wide scope, and allowing a multidisciplinary approach.

### Data collection

#### Medical

In the SMILE study, 32 general practitioners (GPs) working in 9 primary health care centers of the Eindhoven Corporation of Primary Health Care Centers are participating.

The GPs register health related data on all their patients using their electronic medical records (EMR) (Medicom^®^). The GP registration is continuously updated, because registration pertains to daily routine. These data concern disease episodes (coded in ICPC [9]), of which some have a special status as a health problem, prescription of medication (coded in ATC), use of GP services, and referrals to medical specialists and other health care workers. In addition to health related data, a limited set of socio-demographic characteristics is registered: age, sex, educational level, and type of living arrangement. Participating GPs are instructed on coding rules and GPs receive feedback using benchmarking. Twice a year data are transferred to our central database on an anonymous base.

#### Patient Questionnaires

In November 2002 both adolescents and adults received the first questionnaire. Participants receive a questionnaire every year in November. Adolescents and people aged 55 years or older receive an additional questionnaire every year in May.

For adolescents (aged 12–18), a special version of the patient questionnaire was developed, as several concepts required different questioning for adolescents, or the use of words had to be adapted to appeal to adolescents. When reaching the age of 19, the adolescents are included in the adult sample, and will consequently receive the 'adult' version of the questionnaire. The concepts monitored and measured through the patient questionnaires are enlisted in table 1.

**Table 1:** concepts measured in the SMILE questionnaires

	**Adults**	**Adolescents**	**55+**
Gender	X	X	X
Age	X	X	X
Age father/mother		x	
Civil state	X		X
Living arrangement	X	X	X
Length	X	X	X
Weight	X	X	X
Ethnicity	X	X	
Religion	X	X	
Work situation/schoolgoing	X	X	X
Sick leave	X		
Occupational level [20]	X		X
Educational level	X	X	X
Educational level partner			X
Educational level father/mother			X
Income	X	X	X
Appreciation of income [21]			X
Material deprivation [22]			X
Control beliefs: Self-efficacy (Sherer,1982) [23] [24, 25]	X	X	
Control beliefs: mastery [26]			X
Health locus of control (Halfens, 1985) [27]	X	X	
Importance of appearance	X		
Perceived value of health	X	X	
Satisfaction with life domains	X		
Self-esteem (Rosenberg,1989) [28]	X	X	
Values [29]	X		
Coping (UCL) [30, 31]	X	X	
Personality (Big 5) [32]			
Life-events (MAS)	X	X	
Changes due to health problems	X	X	
Perceived limitations due to illness	X	X	
Social support (SSl 12-I) [33, 34]	X	X	
Social network	X	X	
Social inadequacy [35]			X
Hostility [36, 37]			X
Neuroticism [38]			X
Rebeliousness [39]			X
Autonomy			X
Hours of sleep	X	X	
Sunbathing and protection	X	X	
Alcohol	X	X	
Smoking	X	X	
Physical activity (SQUASH) [40]	X	X	
Leisure time activity	X	X	
Nutrition	X	X	
Use of health care facilities	X	X	
Anxiety and depression (HADS) [41–43]	X		X
Depression (CES-D) [44, 45]		X	
Psychosomatic complaints (VOEG) [46, 47]	X	X	
Locomotor complaints (Hildebrandt)	X	X	
Pregnancy	X		
Fatigue (CIS) [48]	X	X	
Self-rated health	X	X	
Quality of life [49]	X	X	
Health insurance	X		
Attitude towards health financing	X		
Attitude towards the Dutch society	X		
RAND-36 [50, 51]			X
Global limitation indicator			X
ADL (GARS [52]/Frenchai [53])	X		
Quote [54]	X		

For each measurement a selection of concepts is made is such a way that on the one hand time needed to fill in the questionnaire is acceptable, and on the other hand basic concepts are measured on a regular basis and all disciplines involved are consulted at the construction of the questionnaires at each measurement. Until now, patient-reported data were solely collected using paper questionnaires. Currently, the use of the internet for data collection is explored.

#### Data manangement

Data both from the medical records and the patient questionnaires are stored in a relational database (Oracle). Data are available through a web browser. Variables and processes are defined in XML format that can be used to generate queries to access the relational database. The data infrastructure is based on a number of HL7v3 information models, which will be compatible with national data infrastructure. Data will be available on different levels of detail for different persons and organizations, consistent with the conditions stated in the informed consent. Questionnaire data of individual patients can never be provided to GPs. All study data are stored and kept anonymously at Maastricht University.

#### Privacy

Written informed consent allowing matching of self-reported and medical data, as well as allowing matching of own data with data of family members is asked when a patient registers in one of the participating health care centers. Privacy regulations are in agreement with the Dutch legislation. The medical ethical committee of the Maastricht Academic Hospital has approved of the study protocol of the SMILE study. Furthermore, the study was registered at the Dutch Data Protection Authority. When appropriate, additional studies evaluating interventions will be sent to the medical ethical committee for approval.

## Discussion

This manuscript describes a new scientific initiative, the SMILE study: a longitudinal dynamic cohort using both routinely collected medical information from the EMRs in general practice and periodically collected patient information using questionnaires.

To measure health related processes in time, a long-term prospective cohort study is the most convenient study design. A powerful feature of cohort studies is the opportunity to collect both repeated exposure data and repeated outcome data, which provides many analytical opportunities to deduce the effects of random measurement error and to evaluate various hypothesized temporal relations between exposure and outcome [10]. The dynamic nature of many risk factors and their relation in time to disease occurrence can only be captured in the cohort design; this temporal interplay is inherently absent from cross-sectional data [11]. The ability to evaluate multiple endpoints simultaneously within the same population is particularly valuable when there are competing risks and benefits [10].

The SMILE study comprises a dynamic population, contrary to most of the other health related large cohort studies, like the Framingham Heart Study [12,13], or the Maastricht Aging Study; MAAS [14]. Furthermore, other cohorts are often more focused on a specific condition (e.g. cancer [15]) or on a subsample of the population (e.g. elderly people [16]). We believe our approach to be more effective, because of the many analytic opportunities that are created with relatively small additional efforts.

Possible disadvantage of this approach are: difficulties in deciding which (limited set of) concepts to measure. This was, however, solved by intense communication within SMILE.

Because the establishment of a large cohort typically represents a major investment in resources, it is efficient to examine all major health outcomes [10]. Since SMILE has the capacity to measure a wide variety of both exposures and outcomes, we have the opportunity to address a large number of important public health issues. Making maximum use of such a resource requires the involvement of numerous investigators, both because of the volume of the potential issues and because expert knowledge in various substantive areas is required. This is rapidly changing the nature of epidemiologic research, from numerous small fiefdoms to cooperative groups of interacting investigators. Such working relations, although created by necessity, can greatly enhance the level of scientific endeavor and productivity [10]. Despite all the advantages of interdisciplinary studies, some participating researchers and GPs have shown cold feet. It's not easy being interdisciplinary [17]. The SMILE team spends considerable time and energy to keep together and to stimulate the stakeholders of the different disciplines to assure continuous development of the research model and the accompanying studies. However, now that the conceptual framework as well as the practical implementation of data collection is becoming more clear and definite, and an increasing amount of data is available, participation of interested disciplines is clearly improving, showing from a number of accepted and published manuscripts and abstracts.

More extensive use of the data will be stimulated by continuous efforts to maintain and to further improve data quality. In this regards continuous participation of both GPs and patients is of the utmost importance in a longitudinal study. For this type of research the setting of general practice linked to academic departments is especially suitable, covering both an unselected patient population and a broad scope of morbidity. This is especially true in the Netherlands, where all people are enrolled as a patient in a general practice.

Several efforts are made to stimulate participation, like raffling of record tokens among adolescents. Once a year a newsletter is sent to all participants, containing results, interviews etc. to stimulate participants' engagement in the SMILE study. Also, at each measurement a press release is sent out, which is usually elaborated by the local press and posters are distributed in the practices reminding patients to fill in their questionnaire.

GP involvement is stimulated in other ways. First of all registration is as close as possible to daily routine, so that keeping up the EMRs does not take much additional time.

Benchmarking is another tool which can result in better quality of registration (completeness as well as correctness), and which at the same time helps GPs to improve the quality of their care. Finally, the Eindhoven Corporation of Primary Health Care Centers is receiving a reimbursement to compensate for additional efforts in the context of the SMILE study.

There has been a sharp increase in the scientific use of electronic medical records (EMRs), collected on a routinely basis [18]. These routinely collected data can be used for audit, quality improvement, health service planning, and scientific research. Primary care research networks are increasingly seen as an important tool for the future of primary care. Research from such networks will allow policy makers and practitioners to address primary care and public health issues from the perspective of data rather than mere belief. Stange [19] even stated that research in practice-based networks is critical to the future of primary care and is badly needed to guide both practice and health-policy decisions. The SMILE study can further contribute to these developments.

## Competing interests

The author(s) declare that they have no competing interests.

## Authors' contributions

Marjan van den Akker participated in the design of the study and drafted the manuscript. Mark Spigt critically revised the manuscript. Lore de Raeve contributed to the design of the questionnaire and critically revised the manuscript. Ben van Steenkiste participated in data collection and critically revised the manuscript. Job Metsemakers contributed to the conception of the study and participated in its design and coordination. Ernst van Voorst was responsible for data acquisition and GP management. Hein de Vries conceived of the study and participated in its design and coordination. All authors have approved of the final version of this manuscript.

## Pre-publication history

The pre-publication history for this paper can be accessed here:


